# Effects of Palliative Care Training Program on Knowledge, Attitudes, Beliefs and Experiences Among Student Physiotherapists: A Preliminary Quasi-experimental Study

**DOI:** 10.4103/0973-1075.78449

**Published:** 2011

**Authors:** Senthil P Kumar, Anand Jim, Vaishali Sisodia

**Affiliations:** Department of Physiotherapy, Kasturba Medical College (Manipal University), Mangalore, India; 1 Bethany Navajeevan College of Physiotherapy, Trivandrum, Kerala, India; 2 Career Institute of Medical Sciences, Bhopal, India

**Keywords:** Training effectiveness, Professional training, Educational intervention, Curriculum development, Program evaluation

## Abstract

**Background:**

Physiotherapists play an inherent role in the multidisciplinary palliative care team. Existing knowledge, attitudes, beliefs and experiences influence their team participation in palliative care.

**Aims:**

The objective of this study was to assess the changes in knowledge, attitudes, beliefs and experiences among student physiotherapists who attended a palliative care training program.

**Settings and Design:**

Preliminary quasi-experimental study design, conducted at an academic institution.

**Materials and Methods:**

Fifty-two student physiotherapists of either gender (12 male, 40 female) of age (20.51±1.78 years) who attended a palliative care training program which comprised lectures and case examples of six-hours duration participated in this study. The study was performed after getting institutional approval and obtaining participants’ written informed consent. The lecture content comprised WHO definition of palliative care, spiritual aspects of life, death and healing, principles, levels and models of palliative care, and role of physiotherapists in a palliative care team. The physical therapy in palliative care-knowledge, attitudes, beliefs and experiences scale (PTiPC-KABE Scale)- modified from palliative care attitudes scale were used for assessing the participants before and after the program.

**Statistical Analysis:**

Paired *t*-test and Wilcoxon signed rank test at 95% confidence interval using SPSS 11.5 for Windows.

**Results:**

Statistically significant differences (*P*<0.05) were noted for all four subscales- knowledge (7.84±4.61 points), attitudes (9.46±8.06 points), beliefs (4.88±3.29 points) and experiences (15.8±11.28 points) out of a total score of 104 points.

**Conclusions:**

The focus-group training program produced a significant positive change about palliative care in knowledge, attitudes, beliefs and experiences among student physiotherapists.

## INTRODUCTION

Palliative care involves an integrated multidisciplinary collaborative teamwork of patients, their families, health professionals and general public toward a continuum of care emphasizing on physical, mental, social, spiritual and emotional aspects of care for life-limiting or life-threatening conditions. Education and training in palliative care influences not only the level of care provided but also the level of team participation of the healthcare professionals. Training in palliative care is a challenging process both for the trainers and for the trainees since a real-life scenario can never be simulated in an educational environment. Stating that multidisciplinary or interdisciplinary approach is central to palliative care, Fineberg *et al*.[1] opined that the most commonly represented or core professions in palliative care team include medicine, social work and nursing, though occasionally the clergy and other healthcare professions are also included. Of the other healthcare professionals who are involved in providing palliative care, physical therapists play an integrated role in multi-disciplinary palliative care team right from symptom control to improving quality of life in patients with life-limiting diseases and conditions as reported by Kumar and Jim[2]. Toot[3] explained the interventions provided by physical therapists in hospice and palliative care that may be directed to three facets: 1) delivering direct patient care, 2) educating the patient-family care unit and fellow health professionals, and 3) functioning as a team member.

Patient’s experiences and self-perceived satisfaction was enhanced by physical therapy and this was reported by Dahlin and Heiwe[4] in their survey of palliative care cancer patients where the authors commented as follows;

Physical therapy was important as it enabled independence, provided relief from distressing symptoms, and offered support. However, communication and coordination within the palliative care team has to be improved to minimize the negative impact of symptom distress on patient well-being and quality of life. Also, physical therapists must develop strategies for patient empowerment and methods for assessing and evaluating qualitative aspects of physical therapy in palliative cancer care.

According to Glazer-Waldman *et al*.,[5] the attitudes of health educators toward health and health care are important to the delivery of a high quality of medical care. In the area of health promotion and disease prevention, physical therapists’ primary goal is a change in patients’ behaviors. The acquisition and dissemination of knowledge is important in delivery of adequate level of care. The ability to disseminate knowledge and influence patients’ attitude and behavior change depends on both the physical therapist’s beliefs and behaviors. As pointed out by Sobush and Fehring[6] if patients have to achieve optimal levels of function, physical therapists should examine the suitability of their attitudes, appearances, and behaviors in eliciting desirable outcomes. Physical therapists must present attitudes and behaviors that demonstrate positive health habits if they have to serve as good role models for their patients.

Previous studies that evaluated effects of training in palliative care utilized the educational program as part of a curriculum development among other healthcare professionals like critical care medicine trainees,[7] medical residents,[8] post-graduates in palliative care program Master of Science graduates,[9] and physicians treating sickle cell disease,[10] Such evaluation of curricular content was proposed to be done using palliative education assessment tool (PEAT) developed by Meekin *et al*.[11] which was then applied on evaluating medical schools by Wood *et al*.[12]

Effects of education and training programs on knowledge, attitudes, beliefs and practice were studied by other authors in the fields of nursing and child health.[13,14] In physical therapy, Balogun *et al*.[15] studied student’s attitudes and knowledge following a five-hour education program on AIDS which showed positive benefits on understanding patients but not on their willingness to work with such patients, in contrast to earlier work of Held[16] who found improved willingness following an educational program. To our knowledge, there was no earlier study that evaluated the effectiveness of palliative care training program among physical therapists.

Existing knowledge, attitudes, beliefs and experiences influence their team participation in palliative care. The objective of this study has been to assess if any change that occurred in knowledge, attitudes, beliefs and experiences among physical therapy students who attended a 6-hour educational training program on palliative care.

## MATERIALS AND METHODS

The study ethical conduct was approved by the Institution and written informed consent was obtained from all participants prior to commencement of the study.

Study design: This study used a pre-post quasi-experimental design, in which data were collected from a convenience sample of physical therapy students who attended a formal training program on palliative care and role of physical therapy.

Participants: Final year physical therapy students of the institution attended upon free registration. The physical therapy curriculum had four years of academic training followed by six months of internship in undergraduate degree program. The students had previous experience of evaluating and treating pain in their third and early fourth year of academics during their clinical postings in multispeciality government hospital focused in treatment of cancer patients. Participants were given an initial description and instructions on filling the questionnaires by a qualified physical therapist.

Training program: The training program was an elective educational intervention which comprised a lecture, case examples and active demonstration which consisted of WHO definition of palliative care, spiritual aspects of life, death and healing, principles, levels and models of palliative care, and role of physiotherapists in a palliative care team- for a total duration of six hours, where direct contact training of physiotherapists in palliative care emphasizing on introduction to palliative care, principles and models of assessment and care of persons at hospice, supportive, palliative, end-of-life and bereavement perspectives, goal planning and strategic implementation of physical therapy treatment methods in palliative care was done. Interaction duration of 15 min was allowed both between participants and also with the invited speaker (first author), who was a qualified physical therapist, experienced in pain relief methods for seven years.

The training program required free registration of the participants and was conducted annually on a not-for-profit basis by the institution. The training program is one of its kinds listed in International Association for Hospice and Palliative Care website.[17]

Outcome measurement: The data was collected pre and post-training through a self-administered questionnaire- physical therapy in palliative care-knowledge, attitudes, beliefs and experiences scale (PTiPC-KABE Scale). The scale is a 37-item self-report measure consisting of both qualitative and quantitative data. The full version of the questionnaire is given in appendix-1. The PTiPC-KABE scale was modified from palliative care attitudes scale used earlier by Kain *et al*.[18] on critical care nurses. This questionnaire consisted of short-answers and multiple-choice questions relating to the respondent’s perception of the existent level of knowledge, the existent level of attitudes, the existent level of beliefs, and the existent level of experiences about palliative care.

The PTiPC-KABE scale was was pilot-tested for test-retest reliability and was then used for assessing the participants before and after the program as a primary outcome measure. To avoid bias and to maintain participant anonymity, coding and decoding during data mining and analysis was done by another independent blinded physical therapist.

Evaluation of test-retest reliability of PTiPC-KABE scale: For this purpose, we randomly selected 24 physical therapy students by Lots method from the same sample and we administered the questionnaire twice with a time-gap of 1 h, 2 h before the commencement of the actual study.

Participants who could not understand English, who refused consent or who returned incomplete questionnaires were excluded from analysis. Completed questionnaires were collected by another qualified physical therapist, who was blinded to the study. The data were initially mined and entered for analysis by another blinded physical therapist.

### Data analysis

Evaluation of test-retest reliability was done using intra-class correlation coefficient (ICC) for the total scores of two trials of the PTiPC-KABE scale. A mixed-methods model of primary analysis was done to compare pre-post scores using paired *t*-test and Wilcoxon signed rank test. Test for normality was done using Kolmogorov-Smirnof test. All analyses were done using SPSS 11.5 for windows at 95% confidence interval.

## RESULTS

Of the total 82 physical therapy students- who attended the training program, 76 provided consent and were willing to participate in this study. 24 filled for the initial test-retest reliability of the scale (ICC=.80-.90) for the total score and the rest 52 were considered for this pre-post study. A total of 52 participants- 12 male (23.1%) and 40 female (76.9%) with age 20.51±1.78 years- filled the questionnaire twice- before the commencement of training program and after the completion of the training program.

Statistically significant (*P*<.05) changes post-program was observed for all items of the PTiPC-KABE scale after we adjusted for acquiescence bias of Likert scaling. We combined the moderately agree and strongly agree into “agree” and same for disagree option. No response was given a score of “zero”, disagree was given “one”, neutral/ unsure was given “two” and agree was given “three”. Such a scoring helped us to have a continuous data for computation purposes. We also inverted the scores for the items with negative impact on palliative care such as items 10, 17, 24, 27-33. Thus a total score can thus be derived after adding all the raw scores.

There was a statistically significant change in knowledge (7.84±4.61 points), attitudes (9.46±8.06 points), beliefs (4.88±3.29 points) and experiences (15.8±11.28 points) subscales of PTiPC-KABE scale when analyzed using paired *t*-test [Table 1].

**Table 1:** Pre-post comparison of knowledge, attitudes, beliefs and experiences in palliative care analyzed using paired *t*-test

Subscales	Preprogram (Mean±SD)	Postprogram (Mean±SD)	Level of significance
Knowledge of palliative care	.69±1.08	7.34±4.18	.000*
Attitudes toward palliative care	-2.11±6.25	7.15±4.23	.000*
Beliefs about palliative care	-6.00±2.4	-1.11±1.63	.000*
Experiences in palliative care	-4.69±4.30	11.11±11.68	.000*

* significant at *P* < 0.05 level

**Figure 1:**
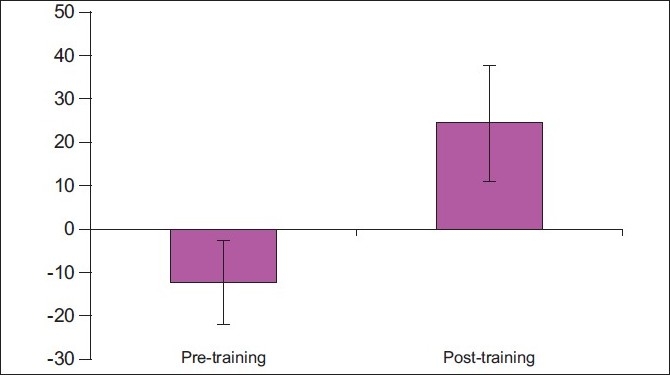
Pre-post comparison of total scores of PTiPC-KABE scale among the study group

There was an overall statistically significant change in total score of 36.61±14.35 points out of a total of 104 points (35.2% change) when analyzed using paired *t*-test [Figure 1].

## DISCUSSION

Our study is the first of its kind reported on physiotherapist population and effects of a palliative care training program. One of the positive consequences of such training programs is the development of curricular content to suit student needs. Such educational interventions pave way to develop better curricular models and to integrate into regular professional and post-professional education.

Marion *et al*.[8] described the principles of integrating palliative care into critical care education which might well be applicable to physical therapy. The authors did a comprehensive review and published consensus which comprised principles, knowledge, attitudes and skills of palliative care.

### Principles

Goals of care should guide use of technology; and decision-making should be patient- centered.

### Knowledge

Know how to use prognostic scoring systems and recognize their limitations; be familiar with the ethical principles and guidelines for forgoing life-sustaining treatments; know how to withdraw life-sustaining treatments in ways that avoid distress; know how to monitor, identify and treat symptoms of distress, discomfort, anxiety or pain; and, know how to use opiates and sedatives to titrate to effect.

### Attitudes

Respect patients as individuals with diverse preferences; value the role of families in shared decision-making; respect ethnic and religious differences; and, be aware of their own values regarding death.

### Skills

Listen and communicate effectively and empathically; form trusting relationships with patients and families; explain details of illness, treatment strategy and goals of care simply; collaborate with the patient and family in balancing benefits and burdens; make recommendations and decisions in the face of uncertainty; give advice based on the patient’s preferences; talk comfortably about death, dying and loss; support families or other intimates during their bereavement; and, work effectively in collaboration with multidisciplinary teams.

We see these- knowledge and attitudes among physical therapists largely shown to influence their practice patterns and levels of care. Earlier reports by Weed and Zimny,[19] Zimny and Tandy[20] showed that improved knowledge leads to enhanced understanding of patient’s presenting problem which in turn leads to high levels of skill in clinical decision-making in physical therapy care of the patient. Battie *et al*,[21] commented that treatment preferences of physical therapists were found to largely vary depending upon not only on the treatment settings but also largely on their perceived attitudes toward treatment methods and their effectiveness.

Population-based interventions are much better as educational interventions aimed at team training in palliative care. Sato *et al*.[22] found population-based educational intervention of 1-h educational lecture focusing on end-of-life home care, life-prolongation treatment and knowledge about palliative care had less long-term positive health outcomes. Solomon *et al*.[23] showed positive impact on their teaching skills, self-awareness, personal understanding of HIV, confidence in teaching, and everyday life when people living with HIV or AIDS (PHA) were considered as healthcare educators. Learner group which included physiotherapy students in their feedback indicated that they valued their interactions with the PHAs. Such an education model would benefit patients and healthcare providers as well.

The study had few limitations- the lack of established validity of the questionnaire used and lack of long-term follow-up of participants to observe knowledge translation into action process. We understand that measurement error of the said questionnaire would be nullified in pre-post designs if the scale has appreciable test-retest reliability. Accordingly the test-retest reliability was excellent. Further research on validation is in progress. Long-term carry-over effect of gained knowledge and its implication into actual real-life situation in palliative care is also under scrutiny currently. Long-lasting improvements in knowledge was found to be absent among medical and nursing students education previously by Velayuthan *et al*.[24] who also suggested that thought should be given to curricular content, teaching methods and evaluation techniques.

The implementation of the above suggested findings, however, face their own barriers. Lai *et al*.[25] listed the currently faced problems in hospice palliative care education which included: (1) The lack of a systematic plan focusing on hospice palliative care and terminal care in schools; (2) The absence of comfort care, communications, ethics, and other relevant issues in extant education and training; (3) The limited number of institutes that currently provide in-service training; (4) The shortage of teachers proficient in both hospice care knowledge and practice; and (5) The current overdependence on traditional education models, which hinders student originality and delays staff growth.

There are already well-established multidisciplinary palliative care training centers for healthcare professionals in operation in Kerala as described by Seamark *et al*.,[26] to name a few: the Calicut centre; the Shanti Avedna Ashram, Mumbai and the Cipla Centre. The Cipla centre had also extended its activities to training medical and nursing colleges in India. It is now up for physiotherapy institutions to take up initiatives to meet the challenges and convert them into educational opportunities if we have a social responsibility as a member of healthcare team.

According to Fins and Nilson,[27] as academic medicine becomes more interested in palliative care education, it is critical that we develop initiatives to overcome attitudinal barriers toward end-of-life care that may undermine learning-and healing.

Considering that physical therapists have largely favorable attitudes toward evidence-based practice as reported by Jette *et al*.[28] and a tremendous volume of evidence supports application of physical therapy in palliative care[2] and a wide scope for physical therapy practice witnessed by a therapist to patient ratio of 1:212,000 in India Higgs *et al*,[29] there is still lot of further scope for development in this physical domain of palliative care in aspects of education, research and practice. In this respect, educational interventions aimed at healthcare professionals tend to bring up an initial favorability toward change in attitudes due to enhanced knowledge thus facilitating positive beliefs and encouraging real life experiences Meier *et al*.[30] This knowledge-attitude-belief-experience is a very important aspect of learning of which the authors have addressed predominantly the first phase of this process. Future studies may address these issues, and include practical skill-oriented training programs with long-term follow-up evaluations.

Physical therapists, let’s now move on, from ignorance to knowledge.[31]

## CONCLUSION

The focused training program on palliative care had positively influenced a group of physiotherapy students in this study by bringing about a significant change in their knowledge, attitudes, beliefs and experiences.

## Appendix: I

**Table:**

Hope you have signed the written informed consent form on the above section.						
Please take your valuable time and fill this questionnaire. The first seven questions are on your personal information (which will never be revealed) and the questions that follow assess you current level of knowledge and your perceived attitudes to palliative care as a physiotherapist. In case of any unseen circumstances, please provide your most suitable answer for your response to as if the situation really occurred in your presence. Kindly select only one option out of the five for each of the items (8-26). Thanks for filling this questionnaire.						
1.	Name:	2. Age:					
3.	Gender:	4.Occupation:				
5.	Contact address:	E-mail ID			Phone:	
6.	Please state your overall interest towards “palliative care”					
7.	Which of the following descriptions (please tick only one) best suit your current position?					
	□ Under-graduate (BPT) student					
	□Physiotherapy intern					
	□Practising physiotherapist,					
	□Post-graduate (MPT) student					
	□Teaching physiotherapist- Tutor, Asst Lecturer, Lecturer, Asst professor, Assoc professor, Professor				
	□Physiotherapy researcher					
	□Doctoral (PhD) student					
	□Physiotherapy department head/ institution head					

		**Strongly disagree**	**Somewhat disagree**	**Neutral (not sure)**	**Somewhat agree**	**Strongly agree**

8.	Palliative care is as important as curative care in physiotherapy practice					
9.	I have had experience of providing palliative care to dying patients and their families					
10.	I feel a sense of personal failure when a patient dies					
11.	There is support for physiotherapy in palliative care in the society					
12.	The medical staffs support physiotherapy in palliative care in my unit					
13.	The physical environment of my unit is ideal for providing palliative care to dying patients					
14.	My unit is adequately staffed for providing the needs of dying patients requiring palliative care and their families					
15.	In my unit, parents/ relatives are involved in decisions about their dying patients					
16.	My previous experiences of providing palliative care to dying patients have been rewarding					
17.	When patients are dying in my unit, providing pain relief is a priority for me					
18.	I am often exposed to death in the physiotherapy department					
19.	Palliative care is necessary in physiotherapy education					
20.	When a patient dies in my unit, I have sufficient time to spend with the family					
21.	There are policies or guidelines to assist in the delivery of palliative care in my unit					
22.	In my unit, when a diagnosis with a likely poor outcome is made, parents/ relatives are informed of palliative care options					
23.	In my unit the team expresses its opinions, values and beliefs about providing care to dying patients					
24.	Caring for dying patients is traumatic for me					
25.	I have received education that assists me to support and communicate with parents/ relatives of dying patients					
26.	All members of the healthcare team in my unit agree with and support palliative care when it is implemented for a dying patient					
27.	In my unit, the staff go beyond what they feel comfortable with in using technological life support					
28.	In my unit, staff are asked by parents/ relatives to continue life-extending care beyond what they feel is right					
29.	My personal attitudes about death affects my willingness to deliver palliative care					
30.	Palliative care is against the values of physiotherapy					
31.	When a patient dies in my unit, counseling is available if I need it					
32.	There is a belief in society that patients should not die, under any circumstances					
33.	Curative care is more important than palliative care in the physiotherapy practice environment					
34.	What do you mean by the term “palliative care”?					
35.	List down any five disease conditions which require “palliative care”					
	1.					
	2.					
	3.					
	4.					
	5.					
36.	Which one of the following is the most important emphasis of “palliative care“?					
	Mobility, functional independence, quality of life. Structural alignment, musculoskeletal endurance, quality of life. Mobility, cardiorespiratory endurance, quality of life. Functional independence, structural alignment, cardiorespiratory endurance.					
37.	State TRUE or FALSE for the following statements:					
	Palliative care provides relief from pain and other distressing symptoms; Palliative care considers life and regards dying as a complicated process; Palliative care intends to hasten or postpone death; Palliative care integrates the psychological and spiritual aspects of patient care; Palliative care offers a support system to help patients die as early as possible; Palliative care offers a support system to help the family cope during the patients illness and in their own bereavement; Palliative care uses a one-one approach to address the needs of patients and their families, including bereavement counselling, if indicated; Palliative care will not enhance quality of life, and does not influence the course of illness; Palliative care is applicable early in the course of illness, in conjunction with other therapies that are intended to prolong life, such as chemotherapy or radiation therapy, and includes those investigations needed to better understand and manage distressing clinical complications.					
